# A pilot randomised trial comparing a mindfulness-based stress reduction course, a locally-developed stress reduction intervention and a waiting list control group in a real-life municipal health care setting

**DOI:** 10.1186/s12889-020-08470-6

**Published:** 2020-03-30

**Authors:** Lise Juul, Karen Johanne Pallesen, Mette Bjerggaard, Corina Nielsen, Lone Overby Fjorback

**Affiliations:** 1 Department of Clinical Medicine, Danish Center for Mindfulness, Aarhus University, Gudrunsvej 78, 3, 8220 Brabrand, Denmark; 2 Vejle Fjord Rehabilitation, Sanatorievej 27 B, 7140 Stouby, Denmark

**Keywords:** Feasibility studies (MeSH), Pilot Projects (MeSH), Pragmatic Clinical Trial (MeSH), Community Mental Health Services (MeSH), Stress, Psychological (MeSH), Mindfulness-based Stress Reduction, MBSR, Mindfulness, Effectiveness, Abbreviations, ACT Acceptance and commitment therapy, ARSQ The Amsterdam Resting State Questionnaire, BRS Brief resilience scale, CI Confidence interval, EQ Experiences questionnaire - decentering sub scale, FFMQ The five facet mindfulness questionnaire, LSR Locally developed stress reduction intervention, MBSR Mindfulness-based stress reduction, PSS Perceived stress scale, RCT Randomised controlled trial, SCL-5 Hopkins Symptom Check List-5, SCS Self-compassion scale, WHO World Health Organization, WHO-5 WHO-5-wellbeing scale

## Abstract

**Background:**

The purpose of the present study was to conduct a pilot randomised controlled trial (RCT) to lend support to a larger effectiveness RCT comparing Mindfulness-Based Stress Reduction (MBSR), a locally-developed stress reduction intervention (LSR) and a waiting list control group in a Danish municipal health care center setting.

**Methods:**

A three-armed parallel pilot RCT was conducted among 71 adults who contacted a Danish municipal health care center due to stress-related problems. Recruitment was made between January and April 2018 and followed usual procedures. Exclusion criteria: 1) acute treatment-demanding clinical depression or diagnosis of psychosis or schizophrenia, 2) abuse of alcohol, drugs, medicine, 3) pregnancy. Randomisation was performed by an independent data manager using the REDCap electronic data capture tool. The primary outcome was a description of RCT feasibility (recruitment and retention rates regarding intervention participation and 12-week follow-up). Secondary outcomes were completion rates regarding questionnaire data and proposed effect-estimates of outcome measures considered to be used in the following real RCT. Type of intervention and outcome assessment were not blinded.

**Results:**

We recruited 71 of 129 eligible individuals from the target population (55, 95%CI: 46–64). Forty-two (59%) were females. Median age: 44 years (1-quartile:34, 3-quartile:50). Twenty-nine (41%) had < 16 years of education. Forty-eight (68%) were employed; 30 of these 48 (63%) were on sick leave. Mean scores for perceived stress (PSS): 25.4 ± 5.3; symptoms of anxiety and depression (SCL-5): 2.9 ± 0.6, and well-being (WHO-5): 31.7 ± 8.5 indicated a need for intervention. 16/24 (67, 95%CI: 45 to 84) who were allocated to MBSR and 17/23 (74, 95%CI: 52 to 90) who were allocated to LSR participated in ≥5 sessions. The loss to follow-up at 12 weeks: MBSR: 5 (21% (95% CI: 7 to 42), LSR: 5 (22% (95% CI: 7 to 44) and waiting list: 4 (17% (95% CI: 5 to 37). This was acceptable and evenly distributed. The results indicated MBSR to be superior.

**Conclusions:**

An RCT assessing the effectiveness of stress reduction interventions in a real-life municipal health care setting is feasible among adults with a clear need for stress reduction interventions based on scores on mental health.

**Trial registration:**

ClinicalTrials.gov. Identifier: NCT03663244. Registered September 10, 2018.

## Background

Mental health problems in general and stress-related issues in particular are of major public health concern. According to the World Health Organization (WHO), stress is one of the major sources of disease [1]. There is considerable evidence that long-term stress increases the number of psychological difficulties and causes physical impairment [2–5]. Furthermore, stress is an independent risk factor for illness and even mortality [5–7]. A Danish population-based study has shown a dose-response relationship between perceived stress measured by the Perceived Stress Scale [8] and mortality within a four-year period [7]. Since the conduction of this study, Danish national health profiles have shown increased levels of self-reported stress [9]. These facts indicate that perceived stress without a clinical diagnosis is an important public health issue to address. The Danish health care system is primarily tax-financed and all citizens have free access to health care. Danish municipalities are responsible for health promotion, prevention and rehabilitation. Thus, many municipal health care centers offer free interventions to citizens with stress-related problems. In the scope of prevention, it is not a requirement to have a clinical diagnosis in order to receive preventive care in the municipal health care centers. The content of the municipal-delivered interventions varies considerably and evaluation of the interventions is rare. Appropriate use of public funding is to apply evidence-based practices. The goal of evidence-based practice is to offer interventions and services based on the highest level of evidence [10, 11]. Mindfulness-Based Stress Reduction (MBSR) is an evidence-based intervention to support participants’ innate resources to cope with stress and challenges in life [12]. MBSR is a well-defined, curriculum-based and replicable group-based intervention lasting 8 weeks [13] as well as a well-described teacher training programme [14]. Research groups from the USA, Europe, Asia and Australia have evaluated MBSR in several randomised controlled trials (RCTs) among individuals with or without a mental or somatic diagnosis [15]. De Vibe et al. conducted a systematic review and a meta-analysis on the effect of MBSR on mental health across a number of outcome measures [15]. A positive effect was shown for different target groups and in a variety of settings. The effect was moderate when compared to a waiting list or a treatment-as-usual control group and smaller, though statistically significant, when compared to an active control group [15]. This review by de Vibe et al. also showed that MBSR also had an effect on somatic outcomes when compared to a waiting list or a treatment-as-usual control group [15]. Khoury et al. have also reported that MBSR had an effect on stress based on a meta-analysis including RCTs in populations without a clinical diagnosis consisting of students, health care professionals or individuals from the background population [16]. Despite the established efficacy, MBSR is rarely applied as stress reduction intervention by the Danish municipalities. A reason could be that the decision makers in the municipal health care centers doubt whether the existing MBSR evidence can be generalised to Danish citizens seeking help due to stress. Participation in MBSR courses in Denmark is currently self-paid. A study has shown that it is mainly highly educated middle-aged women who seek out and pay for an MBSR course in Denmark [17]. Characteristics of participants in stress reduction interventions in Danish municipal health care centers are currently unknown. Dimidjian and Segal have pointed out the lack of effectiveness studies of MBSR [18]. Effectiveness trials differ from efficacy trials by assessing the effect of an intervention under normal conditions e.g. recruiting participants in existing health care settings with established procedures and resources [19]. Effectiveness trials address generalisability and the effect in real-life settings. It is challenging to conduct RCTs in real-life settings because it requires collaboration between institutions such as e.g. universities and municipalities. It is recommended to conduct a pilot trial before the real RCT to assess feasibility and improve the quality of the real RCT [20–22]. The purpose of the present study was thus to conduct a pilot trial to lend support to a larger effectiveness RCT comparing MBSR, a locally-developed stress reduction (LSR) intervention and a waiting list control group in a Danish municipal health care center setting. The LSR intervention is an example of a typical, municipal stress reduction intervention. It was based on the principles of a psychological therapeutic method: the Acceptance and Commitment Therapy (ACT) [23], but the structure and content was developed by the providers, two psychologists in a Danish municipal health care center. The intervention was not manualised, and had not previously been evaluated. The structure and timeframe of LSR were similar to MBSR, though the content and teaching approach were different. Hence, it could also act as a “real-life” active control to take into account the Hawthorne-effect.

Our objectives were to assess 1) the acceptance of trial participation among the target population, including a description of the participant characteristics and recruitment; 2) acceptance of allocated interventions; 3) the degree of contamination (i.e. potential participation in (other) stress reduction treatment interventions in addition to the allocated intervention or non-intervention); 4) the risk of selection problems or bias: loss to 12-week follow-up in the trial arms; 5) the extent of missing data leading to missing outcomes, and 6) indications of potential effects.

## Methods

### Design

We conducted a three-armed parallel pilot RCT in the Municipality of Aarhus in Denmark (340,421 inhabitants) and intended to include 24 participants in each trial arm, in total 72 participants. We assessed that this sample size was justified to meet the objectives of our pilot trial [24]. The inclusion criteria were wide as reflecting the real-life setting. Participants had all contacted Aarhus Municipal Health Care Center due to stress-related problems. Furthermore, participants had to be 18 years or older and able to understand, speak, and read Danish. Exclusion criteria were: 1) acute treatment-demanding clinical depression or a diagnosis of psychosis or schizophrenia, 2) abuse of alcohol, drugs, medicine, and 3) pregnancy.

### Process of the study

The municipal health care center referred all individuals who contacted Aarhus Municipal Health Care Center with stress-related problems between January and April 2018 to the researchers in the project at Aarhus University. A member of the project team contacted each individual by telephone and gave information about the project according to a written guideline. Using a web-based booking system, individuals who met the inclusion and exclusion criteria had an appointment for baseline measurements. They also received detailed written information about the project via e-mail. At the appointment for baseline measurements, a member of the project team also verbally informed the individuals about the project to make sure that individuals fully understood the implications of participation. All attendants at the appointment for baseline measurements agreed to participate and provided written consent. A project team member collected baseline measurements and randomisation was performed. Data collection and randomisation were conducted using the REDCap electronic data capture tools hosted by Aarhus University. REDCap (Research Electronic Data Capture) is a secure, web-based application designed to support data capture for research studies [25]. An independent data manager had programmed the randomisation algorithm in REDCap. The randomisation was stratified for self-reported history of mental disorder. Previous research indicates that self-reported history of mental disorder is associated with a smaller effect of MBSR [17].

Two different staff members performed the baseline measurements using written guidelines, initiated the randomisation in REDCap and informed participants of the results of the randomisation process. The same staff members collected follow-up measurements. Hence, the data collectors were not blinded for group assignment.

We conducted baseline measurements and randomisation in two rounds defined by time-frames to achieve similar intervention and measurement courses of events. In the first round, baseline measurements were conducted between 12 March 2018 and 23 March 2018, interventions between 10 April 2018 and 29 May 2018, and 12-week measurements were conducted between 4 June 2018 and 15 June 2018. In the second round, baseline measurements were conducted between 3 April 2018 and 16 April 2018, interventions between 25 April 2018 and 20 June 2018. Twelve-week measurements were conducted between 18 June 2018 and 3 July 2018. All measurement were made at baseline and at 12 weeks.

### Interventions

**Fig. 1:**
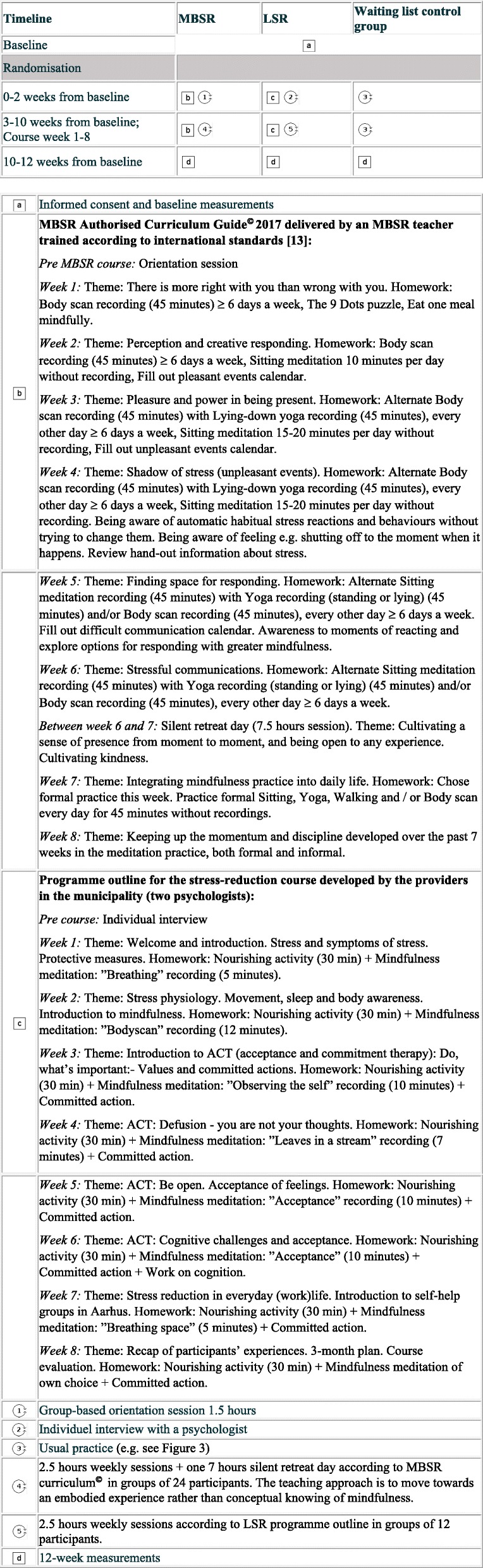
Timeline and description of research activities including intervention contents in a three-armed pilot RCT among individuals seeking help due to stress in a Danish Municipal Health Care Center, 2018. RCT: Randomised controlled trial; MBSR: Mindfulness Based Stress Reduction; LSR: Locally developed stress reduction intervention; ACT: Acceptance and Commitment Therapy. Squares reflect fixed elements and circles reflect the activities that are flexible. This graphical method was proposed by Perera et al. [26]

The PaT Plot [26] in Fig. 1 shows research activities in all three randomised groups.

### MBSR

The curriculum- and evidence-based MBSR course can only be delivered with fidelity by a trained MBSR teacher. It consists of 2.5-h weekly group sessions during 8 weeks with one seven-hour silent retreat day and 45–60 min of daily homework for 6 days a week [13]. The orientation session was performed after randomisation. An MBSR teacher from the Danish Center for Mindfulness taught MBSR in the current pilot trial. She was not a member of the research group. She received supervision and had co-taught two MBSR circles with a MBSR teacher from the research group in order to secure MBSR fidelity. At Danish Center for Mindfulness, the current practice is to allow up to 30 participants in each MBSR group in accordance with recommendations [13]. In order to reflect the usual group size of MBSR, which is twice as high as the group size in LSR, participants in the MBSR course participated in groups along with approximately 12 self-paying participants at the Danish Center for Mindfulness at Aarhus University.

### LSR

The LSR elements are not curriculum-based but developed by psychologists employed in the municipality to provide interventions to people experiencing stress-related problems, and the content of the interventions may vary. At the time of this study, LSR was based on the principles of the psychological therapeutic method ACT [23]. It was an eight-week group-based course with 12 participants and weekly 2.5-h sessions. Between the sessions, participants were recommended to spend 45 min daily on homework. Prior to group enrollment, all participants had an individual consultation with the psychologists teaching the LSR. The effect of LSR and inter-therapist variation have not previously been evaluated.

MBSR and LSR have many similarities, such as structure, timeframe, amount of homework and the group format. Both interventions also include mindfulness training as a central component and encourage participants to create a different relationship with difficult experiences. The main differences between the interventions relate to the content of the interventions and the duration of the meditations. Furthermore, in the LSR intervention, emphasis is placed on a more psychoeducational, or cognitive approach, whereas MBSR is mainly practice-based as the aim is to move towards an embodied experience rather than conceptual knowing of mindfulness [27]. We did not monitor intervention fidelity in this study.

### The waiting list group

We offered the participants allocated to the waiting list group to participate in either LSR or MBSR in August of 2018.

To reflect real-life, there were no restrictions for any of the participants in the three trial arms regarding participation in other stress-reduction interventions during the trial period.

### Outcomes

Primary outcomes of this pilot RCT were: 1) recruitment rate, 2) retention rates regarding intervention participation, 3) retention rates regarding follow-up at 12 weeks, and 4) proportions of allocated participants who had participated in other stress reduction interventions during the trial period. Secondary outcomes were: 1) completion and score rates of study questionnaires, and 2) proposed effect estimates of outcome measures considered to be used in the real RCT.

### Self-reported measures

#### The perceived stress scale (PSS)

Cohen’s 10-item perceived stress scale is a self-report measure of subjective stress [8]. It consists of ten questions indicating how often respondents have found their life unpredictable, uncontrollable, and overloaded in the past month. All items are scored on a five-point Likert scale (total sum scores: 0–40), and higher scores indicate higher levels of stress. The instrument has demonstrated good validity and reliability [28–30]. Scores of PSS have been associated with mortality in a dose-response relationship [7]. Cronbach’s α was 0.84 in the present study sample.

#### The Hopkins symptom check List-5 (SCL-5)

SCL-5 is a five-item self-report measure of symptoms of anxiety and depression [31]. All items are scored on a four-point scale, ranging from 1 (not bothered at all) to 4 (extremely bothered). The score is calculated as the average of the five items with higher scores indicating greater symptoms of anxiety and depression. The SCL-5 originates from the 25-item Symptom Check List (SCL) and correlates at r = 0.92 with the SCL. The alpha reliability for the SCL-5 has been found to be 0.85 [32] A SCL-5 score > 2 has been found to predict mental illness as assessed independently by psychiatrists [32]. Cronbach’s α was 0.85 in the present study sample.

#### The WHO-5-wellbeing scale (WHO-5)

WHO-5 is a five-item self-report measure of wellbeing. It consists of five questions indicating the extent to which respondents have been feeling well during the last 2 weeks. Each question is scored on a five-point scale indicating how often respondents have experienced specific feelings. The points are added and multiplied with four, calculating the total score ranging from 0 to 100; higher scores indicating higher level of wellbeing. The WHO-5 wellbeing scale is considered to be a valid measure of the overall wellbeing of respondents [33]. Cronbach’s α was 0.78 in the present study sample.

#### The brief resilience scale (BRS)

The BRS is a six-item self-report measure of resilience [34]. The BRS consists of six questions. All items range from 1 to 5 (total sum range 6–30). A summary score is created that averages across the six items (range = 1–5), with higher scores indicating a greater perceived recovery from stress [34]. The following cut-off points have been suggested: Scores from 1.00–2.99: low resilience; 3.00–4.30: normal resilience; 4.31–5.00: high resilience [34]. Cronbach’s α was 0.82 in the present study sample.

#### The Amsterdam resting state questionnaire (ARSQ)

The ARSQ is a self-report questionnaire to sample thoughts and feelings during rest, i.e., an awake state characterised by the absence of goal-directed cognitive activity. The scale consists of 21 statements scored on a Likert scale from 1 (completely disagree) to 5 (completely agree) [35]. The ARSQ identifies seven dimensions of resting state cognition: Discontinuity of Mind, Theory of Mind, Self, Planning, Sleepiness, Comfort, and Somatic Awareness. During the resting state, the mind typically wanders in a way that represents habitual ways of thinking. MBSR targets those habitual, normally persistent patterns of thoughts and feelings, hence the programme has the potential of inducing change. In the present study sample, Cronbach’s α was: Discontinuity of Mind (0.86), Theory of Mind (0.59), Self (0.46), Planning (0.77), Sleepiness (0.79), Comfort (0.85), and Somatic Awareness (0.71).

#### The self-compassion scale (SCS) - short form

The SCS is a 12-item self-report measure of self-compassion [36]. It originates from the Self-Compassion Scale (SCS) and has shown good internal consistency and a close to perfect correlation with the long SCS [36]. Self-compassion is proposed to be a contributing mediator of the effect of mindfulness [27] and increased self-compassion has been found to mediate the beneficial effect of MBCT on post-treatment symptoms of depression [37]. Cronbach’s α was 0.87 in the present study sample.

#### The five facet mindfulness questionnaire (FFMQ-15)

FFMQ-15 is a 15-item self-report measure of the dispositional tendency to be mindful in daily life [38]. It is developed from the original FFMQ-39 and has been found to be reliable and valid [38]. It consists of five facets of mindfulness including observing, describing, acting with awareness, non-judgment and non-reactivity. Cronbach’s α was 0.86 in the present study sample.

#### The experiences questionnaire (EQ) - decentering sub scale

The EQ - decentering sub scale is a validated 11-item self-report measure of decentering [39]. Decentering refers to the ability to observe thoughts and feelings as temporary and automatic events in the mind, rather than facts or true descriptions of reality. The items of the decentering factor assess three facets: the ability to distinguish one’s self from one’s thoughts, the ability not to automatically react to one’s negative experiences and the capacity for self-compassion. All items are scored on a five-point Likert scale ranging from 1 to 5 and the total sum score ranges from 11 to 55. Cronbach’s α was 0.81 in the present study sample.

### Clinical measures

#### Blood pressure

Brachial blood pressure was measured after a 10-min rest with an automated blood pressure monitor. Three measurements were made and the average of each pressure index constitutes the values of systolic and diastolic blood pressure in mmHg.

#### Weight

Weight was measured twice without shoes in light indoor clothing to the nearest 0.1 kg. The measurements were completed twice, and the average constitutes the value of weight in kg.

#### Waist circumference

Waist circumference was measured with the participant in the standing position at the mid-point between the lower costal margin and the level of the anterior superior iliac crest to the nearest millimeter. The measurements were completed twice, and the average constitutes the value of waist circumference in cm.

The rationale for collecting the clinical outcome data was the allostatic load theory.

The allostatic load theory is a theoretical framework for physiological pathways that may explain relations between mental and physical well-being [40–42]. Furthermore, an allostatic load index has been developed, consisting of both primary mediators of the stress response in conjunction with clinically relevant biomarkers representing secondary outcomes. The index has been found to be a strong predictor of disease development and mortality [43, 44]. Blood pressure, weight and waist circumference are parts of this index.

### Data analysis

We estimated proportions and proposed effect estimates with 95% confidence intervals (CIs). We estimated the participation rates in the two intervention arms using two cut-off points: participation ≥5 sessions and participation ≥7 sessions. Furthermore, we estimated medians with 1st and 3rd quartiles of the total number of sessions. We performed a loss to 12-week follow-up analysis for age, gender, educational level, employment, sick-leave, history of mental disorder, participation in ≥5 sessions of allocated intervention, and pre-scores of PSS, SCL-5, WHO-5 and BRS by t-test and chi_2_-test. We also performed a loss to course participation analysis (participation in < 5 sessions of allocated intervention) for age, gender, educational level, employment, sick leave, history of mental disorder, and pre-scores of PSS, SCL-5, WHO-5 and BRS by t-test and chi_2_-test.

We estimated the mean changes in the mental health and clinical outcomes from baseline to 12-week follow-up in the three groups. We compared the differences in the mean change in the mental health outcomes between the groups using linear regression models adjusting for age, sex, educational level, history of mental disorder and baseline scores of mental health (PSS, SCL-5, WHO-5 and BRS). When we compared the differences in the mean change in the clinical outcomes between the groups, we adjusted for age, sex, educational level, history of mental disorder, baseline blood pressure, body mass index (BMI), weight and waist circumference using linear regression models. We adjusted for the above potential confounders because they were not evenly distributed at baseline across the three groups due to the small study sample. We present proposed effect estimates with 95% CIs without *p* values as suggested by the CONSORT statement, as pilot trials are not powered for testing hypotheses about effectiveness [22]. Bonferroni correction should be considered in a real effectiveness RCT. We did not make analyses to take into account missing data. All analyses were performed in STATA 14.

## Results

**Fig. 2:**
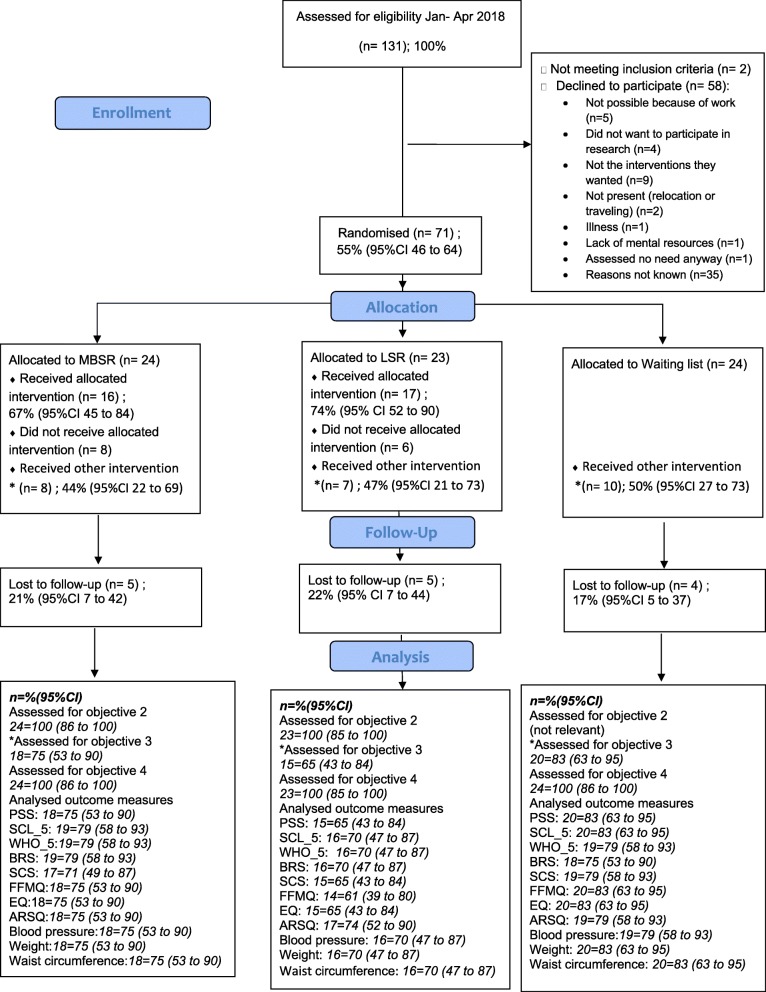
Trial profile for a three-armed pilot Randomised Controlled Trial comparing stress reduction interventions in a Danish Municipal Health Care Center, 2018. RCT: Randomised controlled trial; MBSR: Mindfulness Based Stress Reduction; LSR: Locally developed stress reduction intervention; CI: confidence interval; PSS: Perceived Stress Scale; SCL-5: Hopkins Symptom Check List-5; WHO-5: WHO-5-wellbeing scale; BRS: Brief Resilience Scale; ARSQ: The Amsterdam Resting State Questionnaire; SCS: Self-Compassion Scale; FFMQ: The Five Facet Mindfulness Questionnaire; EQ: Experiences Questionnaire - Decentering sub scale

From 25 January to 10 April 2018, 131 citizens contacted the Aarhus Municipal Health Care Center due to stress-related problems. Two individuals did not meet the inclusion criteria. Among the 129 eligible, 71 individuals (55, 95% CI: 46–64) consented to participate in the RCT. Hence, 71 of the planned 72 participants were included in the RCT within the scheduled time-frame. The characteristics of the study population are shown in Table 1, and the primary results are shown in Fig. 2.

**Table 1:** Characteristics of 71 participants included in a three-armed pilot RCT among individuals seeking help due to stress in a Danish Municipal Health Care Center, 2018

	MBSR (***n*** = 24)	LSR (***n*** = 23)	Waiting list (n = 24)
Gender, female (%)	11 (46)	16 (70)	15 (63)
Age, median (q1,q3)	46 (36, 51)	41 (29, 49)	45 (41, 52)
Education (%)			
≤ 11 years	2 (8)	1 (4)	0
> 11 < 16 years	6 (25)	10 (43)	10 (42)
≥ 16 years	16 (67)	12 (52)	14 (58)
Living alone (%)	5 (21)	4 (17)	5 (21)
Living with parents (%)	0	1 (4)	0
Living with a partner, no children (%)	9 (38)	7 (30)	9 (38)
Living with a partner and children/adolescents (%)	6 (25)	3 (13)	6 (25)
Living with children/adolescents, no partner (%)	2 (8)	4 (17)	2 (8)
Living with other adults (%)	2 (8)	4 (17)	2 (8)
Employment status			
Employed (%)	17 (71) (9 (53%) sick leave)	15 (65) (8(53%) sick leave)	16 (67) (13 (81%) sick leave)
Unemployed (%)	3 (13)	5 (22)	5 (21)
Sick leave > 3 months (%)	2 (8)	2 (9)	2 (8)
Other	2 (8)	1 (4)	1 (4)
Disease, present/earlier (%)			
Asthma	0 / 3 (13)	3 (13) / 1(4)	1 (1) / 1(1)
Diabetes	0 / 0	1 (4) / 0	0 / 0
Hypertension	3 (13) / 1 (4)	3 (13) /3 (13)	3 (13) / 3 (13)
Myocardial infarction	0 / 0	0 / 0	0 / 0
Angina pectoris	0 / 2 (8)	0 / 0	0 / 0
Stroke	0 / 0	0 / 0	0 / 0
COLD	0 / 1 (4)	1 (5) / 0	0 / 0
Osteoarthritis	5 (21) / 0	2 (9) / 0	1 (4) / 1 (4)
Rheumatoid arthritis	2 / (8) / 0	1 (4) / 0	0 / 1 (4)
Osteoporosis	3 (13) / 0	0 / 0	0 / 0
Prolapse	5 (21) / 1 (4)	1 (5) / 2 (9)	1 (4) / 2 (8)
Cancer	0 / 1 (4)	0 / 0	0 / 0
Migraine	7 (29) / 2 (8)	4 (17) / 8 (35)	6 (25) / 4 (17)
Mental disorder ≤6 months	5 (21) / 3 (13)	2 (9) / 1 (4)	2 (9) /1 (4)
Mental disorder > 6 months	4 (17) / 1 (4)	4 (18) / 0	2 (8) /4 (17)
*Self-reported mental health*			
Perceived Stress Scale, mean (SD)	24.6 (5.6)	24.9 (5.6)	26.6 (4.6)
Symptom Check List_5, mean (SD)	2.8 (0.6)	3.0 (0.8)	3.0 (0.5)
Well-being, WHO-5, mean (SD)	37.2 (19.1)	32.7 (16.2)	30.8 (15.2)
Brief Resilience Scale, mean (SD)	3.2 (0.5)	3.2 (0.4)	3.5 (0.6)
Five Facet Mindfulness Questionnaire_15, mean (SD)	45.7 (9.4)	44.5 (7.3)	45.3 (10.6)
Self-compassion Scale, mean (SD)	31.4 (8.8)	30.6 (7.4)	33.2 (9.2)
Decentering EQ, mean (SD)	30.8 (7.5)	30.5 (6.5)	30.7 (7.2)
*Clinical measurements*			
Systolic blood pressure, mmHg, mean (SD)	127.1 (15.1)	125.7 (18.9)	123.4 (17.0)
Diastolic blood pressure, mmHg, mean (SD)	82.6 (9.9)	80.7 (13.1)	81.6 (11.6)
Weight, Kg, median (q1, q3)	79.0 (70.0, 92.0)	75.0 (64.0, 92.0)	68.5 (63.5, 84.0)
BMI, kg/m^2^ ≥ 25, (%)	18 (75)	14 (61)	9 (39)
Waist circumference, cm, median (q1, q3)	95.5 (90.5, 106.5)	94.0 (87.0, 105.0)	92.0 (80.5, 99.0)

*RCT* randomised controlled trial, *MBSR* mindfulness based stress reduction, *LSR* locally developed stress reduction intervention, *CI* confidence interval, *q* quartile, *BMI* body mass index

### Attendance to the interventions

The median number of completed sessions in the MBSR group was six (1-quartile:1, 3-quartile:7.5). The median number of completed sessions in the LSR group was seven (1-quartile: 4, 3-quartile: 8).

A total of 16 of the 24 participants (67, 95% CI: 45 to 84) allocated to MBSR participated in ≥5 sessions. Among the 23 participants allocated to LSR, 17 (74, 95% CI: 52 to 90) participated in ≥5 sessions.

A total of 11 of the 24 participants (46, 95% CI: 26 to 67) allocated to MBSR participated in ≥7 sessions. Among the 23 participants allocated to LSR, 16 (70, 95% CI: 47 to 87) participated in ≥7 sessions.

No statistically significant association was found among participants attending < 5 sessions in either the MBSR course or the LSR intervention. However, there was a tendency that not being on sick leave was associated with participating < 5 sessions (*p* = 0.07).

### Other stress reduction interventions

**Fig. 3:**
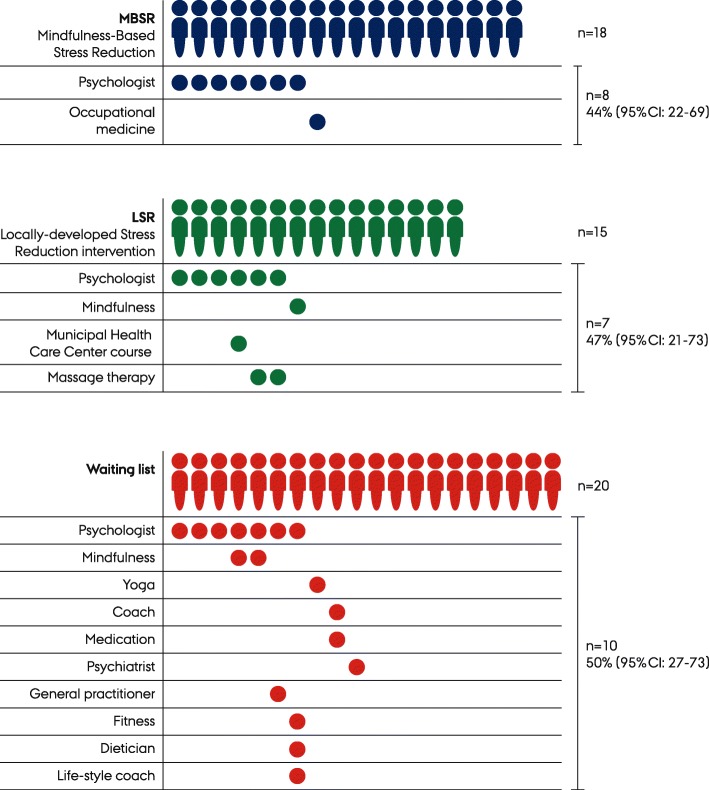
Participation in other stress reduction interventions during the trial period in three randomised groups in a pilot Randomised Controlled Trial comparing stress reduction interventions in a Danish Municipal Health Care Center, 2018

A total of 18 participants in the MBSR group, 15 participants in the LSR and 20 participants in the waiting list group completed the questions about participation in other stress reduction interventions during the trial period (Fig. 3).

### Loss to follow-up

A total of 18 participants in the MBSR group, 16 participants in the LSR group, and 20 participants in the waiting list group attended the 12-week follow-up. Furthermore, one participant from the MBSR group, and two from the LSR group completed the questionnaire sent by e-mail to all non-attenders at follow-up. Hence, the loss to follow-up in the three groups were: MBSR: 5 (21% (95% CI: 7 to 42), LSR: 5 (22% (95% CI: 7 to 44) and waiting list: 4 (17% (95% CI: 5 to 37) (Fig. 2). The only characteristic associated with loss to follow-up was participation < 5 sessions in each allocated intervention.

### Outcome measures

The proportions of completed questionnaire scales among those completing the questionnaires in the three randomised groups are shown in Table 2. Two participants at the most in the MBSR and the waiting list group had missing questionnaire data leading to missing outcomes, whereas up to four participants in the LSR group had missing outcomes due to missing questionnaire data.

**Table 2:** Completed questionnaire scales in a three-armed pilot RCT among individuals seeking help due to stress in a Danish Municipal Health Care Center, 2018

Questionnaire scale	MBSR (*n* = 19) n; % (95%CI)	LSR (*n* = 18) n; % (95%CI)	Waiting-list (*n* = 20) n; % (95%CI)
PSS	18; 95 (74 to 100)	15; 83 (59 to 96)	20; 100 (83 to 100)
SCL-5	19; 100 (82 to 100)	16; 89 (65 to 99)	20; 100 (83 to 100)
WHO-5	19; 100 (82 to 100)	16; 89 (65 to 99)	19; 95 (75 to 100)
BRS	19; 100 (82 to 100)	16; 89 (65 to 99)	18; 90 (68 to 99)
ARSQ	18; 95 (74 to 100)	17; 94 (73 to 100)	19; 95 (75 to 100)
FFMQ	18; 95 (74 to 100)	14; 78 (52 to 94)	20; 100 (83 to 100)
SCS	17; 89 (67 to 99)	15; 83 (59 to 96)	19; 95 (75 to 100)
EQ	18; 95 (74 to 100)	15; 83 (59 to 96)	20; 100 (83 to 100)

*RCT* randomised controlled trial, *MBSR* mindfulness based stress reduction, *LSR* locally developed stress reduction intervention, *CI* confidence interval, *PSS* perceived stress scale, *SCL-5* Hopkins Symptom Check List-5, *WHO-5* WHO-5-wellbeing scale, *BRS* brief resilience scale, *ARSQ* The Amsterdam Resting State Questionnaire, *SCS* self-compassion scale, *FFMQ* the five facet mindfulness questionnaire, *EQ* experiences questionnaire - Decentering sub scale

### Proposed effect sizes in a future RCT

Table 3 shows the 12-week changes as well as indications of the effectiveness of MBSR and LSR compared to the waiting list group. Table 4 shows indications of the effectiveness of MBSR compared to LSR. Proposed effect sizes of outcomes that could be used as mediator outcomes in a real RCT are available in Tables 1a and 2a.

**Table 3:** Indications of effectiveness of MBSR and LSR compared with a waiting list control group 12 weeks from baseline (regression analysis). A three-armed pilot RCT among individuals seeking help due to stress in a Danish Municipal Health Care Center (19 + 20 + 18 in the MBSR, Waiting list and LSR group, respectively), 2018

Change 12 weeks from baseline, mean (95% CI)	Difference compared with waiting list control group, mean (95%CI)	Adjusted^a^ difference compared with waiting list control group, mean (95%CI)
*Self-reported outcomes (score-points)*		
**Perceived Stress (PSS)**		
MBSR: −8.3 (−11.1 to −5.5)	−2.8 (−6.3 to 0.7)	− 2.9 (− 6.7 to 0.8)^b^
WAITING LIST: − 5.5 (− 7.8 to − 3.2)		
LSR: − 6.3 (− 9.4 to − 3.1)	− 0.8 (− 4.4 to 2.9)	− 0.8 (− 5.3 to 3.6)^b^
**Symptoms anxiety and depression (SCL_5)**		
MBSR: − 0.8 (− 1.1 to − 0.5)	−0.2 (− 0.6 to 0.2)	−0.3 (− 0.7 to 0.1)^b^
WAITING LIST: − 0.6 (− 0.8 to − 0.3)		
LSR: − 0.6 (− 1.0 to − 0.2)	−0.01 (− 0.4 to 0.4)	0.06 (− 0.5 to 0.4)^b^
**Well-being (WHO-5)**		
MBSR: 22.1 (12.3 to 31.9)	13.3 (−0.1 to 26.6)	12.9 (1.2 to 25.6)^b^
WAITING LIST: 8.8 (−0.9 to 18.6)		
LSR: 20.0 (10.5 to 29.5)	11.2 (− 2.1 to 24.4)	6.3 (−6.4 to 18.9)^b^
**Resilience (BRS)**		
MBSR: 0.5 (0.2 to 0.8)	0.4 (−0.1 to 0.9)	0.5 (0.1 to 0.9)^b^
WAITING LIST: 0.1 (−0.2 to 0.5)		
LSR: 0.2 (−0.4 to 0.7)	0.0 (−0.5 to 0.6)	0.1 (− 0.5 to 0.6)^b^
*Clinical outcomes*		
**Systolic blood pressure, mmHg**		
MBSR: − 3.6 (− 7.7 to 0.5)	1.3 (−4.0 to 6.6)	1.5 (− 4.3 to 7.3)^c^
WAITING LIST: − 4.9 (− 8.6 to − 1.2)		
LSR: − 4.3 (− 9.7 to 1.0)	0.6 (− 5.5 to 6.7)	4.9 (− 0.2 to 10.1)^c^
**Diastolic blood pressure, mmHg**		
MBSR: − 2.2 (− 5.5 to 2.0)	1.8 (− 2.0 to 5.7)	0.7 (− 3.4 to 5.2)^c^
WAITING LIST: − 4.1 (− 6.5 to − 1.6)		
LSR: − 4.5 (− 7.7 to − 1.4)	− 0.5 (− 4.2 to 3.3)	1.4 (− 2.6 to 5.4)^c^
**Waist circumference, cm**		
MBSR: 1.3 (− 0.8 to 3.4)	5.8 (2.1 to 9.5)	4.6 (0.8 to 8.5)^d^
WAITING LIST: − 4.5 (− 7.6 to − 1.4)		
LSR: − 0.3 (− 3.2 to 2.7)	4.3 (0.1 to 8.5)	5.0 (0.8 to 9.2)^d^
**Weight, Kg**		
MBSR: − 0.3 (− 1.5 to 0.9)	0.4 (− 0.9 to 1.8)	0.8 (− 0.5 to 2.2)^d^
WAITING LIST: − 0.7 (− 1.4 to 0.0)		
LSR: 0.2 (− 1.1 to 1.5)	0.9 (− 0.5 to 2.2)	1.0 (− 0.5 to 2.6)^d^

*RCT* randomised controlled trial, *MBSR* mindfulness based stress reduction, *LSR* locally developed stress reduction intervention, *CI* confidence interval, *PSS* perceived stress scale, *SCL-5* Hopkins Symptom Check List-5, *WHO-5* WHO-5-wellbeing scale, *BRS* brief resilience scale, ^a^Adjusted for age, sex, educational level, history of mental disorder, ^b^ also adjusted for baseline PSS, SCL-5, WHO-5 and BRS, ^c^ also adjusted for baseline systolic and diastolic bold pressure, ^c^ also adjusted for baseline BMI, weight and waist circumference

**Table 4:** Indications of effectiveness of MBSR compared with LSR 12 weeks from baseline (regression analysis). A pilot RCT among individuals seeking help due to stress in a Danish Municipal Health Care Center (19 + 18 in the MBSR and LSR group, respectively), 2018

Change 12 weeks from baseline, mean (95%CI)	Difference, mean (95%CI)	Adjusted^a^ difference, mean (95%CI)
*Self-reported outcomes (score-points)*		
**Perceived Stress (PSS)**		
MBSR: −8.3 (− 11.1 to − 5.5)	− 2.0 (− 6.0 to 2.0)	− 2.2 (− 7.0 to 2.5)^b^
LSR: − 6.3 (− 9.4 to − 3.2)		
**Symptoms anxiety and depression (SCL_5)**		
MBSR: − 0.8 (− 1.1 to − 0.5)	−0.2 (− 0.7 to 0.2)	−0.2 (− 0.6 to 0.2)^b^
LSR: − 0.6 (− 1.0 to − 0.2)		
**Well-being (WHO-5)**		
MBSR: 22.1 (12.3 to 31.9)	2.1 (− 11.2 to 15.4)	4.9 (− 7.3 to 17.2)^b^
LSR: 20.2 (10.5 to 29.5)		
**Resilience (BRS)**		
MBSR: 0.5 (0.2 to 0.8)	0.4 (−0.2 to 0.9)	0.2 (−0.3 to 0.8)^b^
LSR: 0.2 (−0.4 to 0.7)		
*Clinical outcomes*		
**Systolic blood pressure, mmHg**		
MBSR: −3.6 (− 7.7 to 0.5)	0.7 (−5.7 to 7.1)	−0.2 (− 6.3 to 5.9)^c^
LSR: −4.3 (− 9.7 to 1.0)		
**Diastolic blood pressure, mmHg**		
MBSR: −2.2 (−5.5 to 2.0)	2.3 (− 2.0 to 6.7)	3.0 (− 2.0 to 7.9)^c^
LSR: −4.5 (− 7.7 to − 1.4)		
**Waist circumference, cm**		
MBSR: 1.3 (−0.8 to 3.4)	1.6 (−1.8 to 4.9)	0.9 (− 3.3 to 5.1)^d^
LSR: −0.3 (− 3.2 to 2.7)		
**Weight, Kg**		
MBSR: −0.3 (−1.5 to 0.9)	− 0.5 (−2.2 to 1.2)	−0.7 (− 2.3 to 1.0)^d^
LSR: 0.2 (− 1.1 to 1.5)		

*RCT* randomised controlled trial, *MBSR* mindfulness based stress reduction, *LSR* locally developed stress reduction intervention, *CI* confidence interval, *PSS* perceived stress scale, *SCL-5* Hopkins Symptom Check List-5, *WHO-5* WHO-5-wellbeing scale, *BRS* brief resilience scale; ^a^Adjusted for age, sex, educational level, history of mental disorder, ^b^ also adjusted for baseline PSS, SCL-5, WHO-5 and BRS, ^c^ also adjusted for baseline systolic and diastolic bold pressure, ^c^ also adjusted for baseline BMI, weight and waist circumference

## Discussion

### Main findings and comparison with existing literature

The study showed that it was feasible to include more than half of the total target population during a short time frame. We found that the group of self-referred individuals was a highly relevant group with an obvious potential for prevention. This is an important finding as the characteristics of individuals seeking help for stress-related problems in Danish municipal health care centers have not been described previously. Thus, the receivers of current free municipal stress reduction intervention have a clear need based on scores on self-reported mental health and are relevant for inclusion into an effectiveness RCT. The mean PSS score was higher than 18, which has been associated with higher mortality within a four-year period [7]. The mean SCL-5 was higher than 2, which has been found to predict the presence of a mental illness assessed independently by psychiatrists [32]. The mean WHO-5 score at 31.7 ± 8.5 was at a substantially lower level compared to the general population. The Danish Health Authority advices people with a WHO-5 score < 35 to seek help [45]. Finally, the mean score of resilience was in the lower range. As the majority of participants had a job but was absent due to stress, interventions due to stress-related problems may potentially contribute to prevent people from leaving the labour market prematurely. In comparison with the majority of MBSR research, the current study population differed as we included more men and more individuals with a lower level of education [16, 46]. Offering free stress reduction interventions in a municipal health care center reaches a target group with a lower level of education and a clear need for stress management based on scores on self-reported mental health compared to offering self-paid MBSR classes [17].

Both MBSR and LSR seemed to be accepted programmes for the participants as indicated by the number of sessions attended. However, there was a higher proportion of participants within the LSR group that attended almost all sessions in the intervention. Almost half of the participants across the three groups received other stress reduction interventions during the RCT. This may explain why improvements also appeared in the waiting list group. This calls for RCTs to investigate added effectiveness of e.g. municipal-delivered interventions. Individual psychologist consultations were evenly distributed across the groups. It is unknown to which extent participation in MBSR or LSR has evoked seeking more interventions (help / self-care), or whether psychologist consultations were initiated before the trial and continued during the trial. A real effectiveness RCT will uncover questions about the added value of offering MBSR and LSR in a municipal health care center to individuals who seek help due to stress. This is highly important for and relevant to decision makers to ensure the optimal offers in this population.

Dimidjian and Segal in their review did not identify any effectiveness RCT evaluating the effect of MBSR in a community setting [18]. To our knowledge, such a trial has so far not been conducted. Our pilot RCT indicates that it is feasible to conduct an effectiveness RCT evaluating MBSR in a Danish community setting. The loss to follow-up was acceptably low and evenly distributed in the three groups in our study. However, we consider the risk of bias due to more missing questionnaire data among the participants in the LSR group and would maybe apply imputation of missing data in a real RCT. Nyklicek et al. [47] and Robins et al. [48] evaluated effects of MBSR in community residents with stress-related problems in the Netherlands and USA, respectively. Their participants were recruited by advertisement in local papers [47] and by flyers in university and hospital settings [48]. These are usual ways of recruiting participants to traditional efficacy RCTs, but these recruitment procedures do not reflect the real-life health care service access to e.g. free municipal stress reduction intervention. The study populations and the effects in these efficacy RCTs may not be comparable to our context. In the above efficacy trials, there were only very small improvements in the control groups. Our pilot RCT conducted in a Danish municipal setting showed significant improvements on self-reported mental health in all three randomised groups at 12 weeks. This finding may reflect that individuals seek help to find alternatives when allocated to a waiting list, which our study also showed. Some of the improvement may also be natural recovery, well-known as “regression towards the mean”. However, our findings suggest that pre- and post-evaluations in real-life settings should be interpreted with caution and thus highlight the importance of conducting effectiveness RCTs. A pilot RCT is not designed to assess effectiveness. Most often effectiveness RCTs require large sample sizes, because effects will always be diluted in real-life. However, the effect estimates from real effectiveness RCTs are immediately useful for decision makers and health care providers [19]. Furthermore, small effect estimates in effectiveness RCTs often correspond to larger effect estimates in efficacy RCTs and may have a considerable impact at societal level [49].

### Indications of effectiveness

Pilot RCTs like ours can provide estimates of proposed effects in a real effectiveness RCT with CIs pointing to the degree of uncertainty. The interesting point is whether there are indications of differences in outcomes. Interpreted with caution, our results may indicate potential positive effectiveness of MBSR on mental health compared to wait-list and LSR (Tables 3 and 4). Our results showed statistically significant positive effects of MBSR concerning well-being and resilience (Table 3) and on resting state (discontinuity of mind, planning and body awareness) (Additional material Table 1a) compared to the waiting list group. Furthermore, our results quite clearly indicated positive effects of MBSR on stress and symptoms of anxiety and depression compared to the waiting list group (Table 3). The effect estimates of all mental outcomes were in favour of MBSR compared to LSR, however with the 95% confidence intervals, including effect estimates going in both directions (Table 4). The effect estimate of well-being was in favour of LSR compared to wait-list, also with the 95% confidence intervals, including effect estimates going in both directions (Table 3). There were no indications of effects of LSR on stress, symptoms of anxiety, and depression or resilience compared to wait-list controls (Table 3).

We found an unexpected effect of waist circumference reduction in the waiting list group. This finding is unexplained, but may be caused by a measurement error. Baseline and follow-up waist measurements could have been performed by two different staff members, who measured differently despite written guidelines. However, we cannot explain why measurement errors were more pronounced in the waiting list group. No effects on weight were seen between groups. Allocation to the waiting list group may also have evoked behavioural changes. This finding should be explored in a real RCT where more consideration to the precise measurement of waist circumference must be ensured.

### Strengths and limitations

A major strength of this pilot RCT is that it was conducted in a real-life setting in the target population which was supposed to benefit from the intervention. The pilot RCT was conducted with methodological rigor. However, it is a limitation that we do not have socio-demographic data on the individuals who declined participation. It is also a limitation that the staff performing the measurements were not blinded to the randomisation. In the real RCT, measurements and randomisation should ideally be conducted by different individuals in different locations. It is debatable, whether it is possible to entirely blind data collectors. It is not possible to prevent participants from telling about their experiences at follow-up.

## Conclusions

This pilot RCT showed that it was feasible to conduct a valid three-armed RCT among adults with a clear need for stress management based on scores on self-reported mental health in a real-life municipal health care setting. We thus recommend proceeding with a real effectiveness RCT to further strengthen the results of our pilot RCT.

## Supplementary information

**Additional file 1: Table 1a. (Proposed mediators)** Indications of effectiveness of MBSR and LSR compared with a waiting list control group 12 weeks from baseline (regression analysis). A three-armed pilot RCT among individuals seeking help due to stress in a Danish Municipal Health Care Center (19 + 20 + 18 in the MBSR, Wait-list and LSR group, respectively), 2018.

**Additional file 2: Table 2a. (Proposed mediators)** Indications of effectiveness of MBSR compared with LSR 12 weeks from baseline (regression analysis). A three-armed pilot RCT among individuals seeking help due to stress in a Danish Municipal Health Care Center (19 + 18 in the MBSR and LSR group, respectively), 2018.

## Data Availability

The dataset used and analysed during this study is available from the corresponding author by reasonable request.
