# Supplementary material for: A pilot randomised trial comparing a mindfulness-based stress reduction course, a locally-developed stress reduction intervention and a waiting list control group in a real-life municipal health care setting
Source: BMC Public Health. 2020 Mar 30;20:409. doi: 10.1186/s12889-020-08470-6 (PMC7106861; doi:10.1186/s12889-020-08470-6)
Supplement: Supplementary file 1 — Additional file 1: Table 1a. (Proposed mediators) Indications of effectiveness of MBSR and LSR compared with a waiting list control group 12 weeks from baseline (regression analysis). A three-armed pilot RCT among individuals seeking help due to stress in a Danish Municipal Health Care Center (19 + 20 + 18 in the MBSR, Wait-list and LSR group, respectively), 2018. [file 12889_2020_8470_MOESM1_ESM.docx]

**Table 1a (Proposed mediators)** Indications of effectiveness of MBSR and LSR compared with a waiting list control group 12 weeks from baseline (regression analysis). A three-armed pilot RCT among individuals seeking help due to stress in a Danish Municipal Health Care Center (19+20+18 in the MBSR, Waiting list control and LSR group, respectively), 2018.

| Change 12 weeks from  baseline,  mean (95%CI) | Difference compared with waiting list control group, mean (95%CI) | Adjusted^a^ difference compared with waiting list control group, mean (95%CI) |
| --- | --- | --- |
| *Self-reported outcomes (score-points)* |  |  |
| Mindfulness (FFMQ_15) |  |  |
| MBSR: 6.1 (1.5 to 10.7) | 2.2 (-3.5 to 7.8) | 1.3 (-4.8 to 7.4) |
| WAITING LIST: 4.0 (0.2 to 7.7) |  |  |
| LSR: 5.8 (1.8 to 9.8) | 1.8 (-3.6 to 7.2) | 0.1 (-6.0 to 6.2) |
| Self-Compassion (SCS) |  |  |
| MBSR: 7.1 (2.7 to 11.4) | 3.4 (-2.6 to 9.4) | 2.5 (-3.8 to 8.8) |
| WAITING LIST: 3.7 (-0.8 to 8.1) |  |  |
| LSR: 6.7 (2.8 to 10.7) | 3.0 (-2.8 to 8.9) | -0.9 (-7.3 to 5.4) |
| Decentering (EQ) |  |  |
| MBSR: 6.9 (2.4 to 11.5) | 4.1 (-2.0 to 10.2) | 4.0 (-2.7 to 10.6) |
| WAITING LIST: 2.9 (-1.5 to 7.2) |  |  |
| LSR: 3.5 (-0.3 to 7.4) | 0.6 (-5.2 to 6.4) | -4.0 (-10.5 to 2.5) |
| *Amsterdam Resting-State Questionnaire* |  |  |
| Discontinuity of mind |  |  |
| MBSR: -1.2 (-1.7 to -0.7) | -0.8 (-1.5 to -0.1) | -0.9 (-1.6 to -0.2) |
| WAITING LIST:-0.3 (-0.8 to 0.29 |  |  |
| LSR: -0.8 (-1.2 to -0.3) | -0.4 (-1.1 to 0.2) | -0.2 (-0.9 to 0.5) |
| Theory of mind |  |  |
| MBSR: -0.1 (-0.5 to 0.3)  WAITING LIST: 0.1 (-0.4 to 0.6)  LSR: -0.2 (-0.7 to 0.3) | -0.2 (-0.9 to 0.4)  -0.3 (-1.0 to 0.4) | -0.4 (-1.2 to 0.3)  -0.6 (-1.5 to 0.3) |
| Self |  |  |
| MBSR: -0.3 (-0.8 to 0.2)  WAITING LIST: -0.1 (-0.4 to 0.2)  LSR: -0.2 (-0.6 to 0.3) | -0.1 (-0.7 to 0.4)  -0.0 (-0.6 to 0.5) | -0.3 (-0.9 to 0.3)  -0.1 (-0.7 to 0.5) |
| Planning |  |  |
| MBSR: -1.0 (-1.5 to -0.5) | -0.9 (-1.6 to -0.2) | -1.1 (-1.9 to -0.3) |
| WAITING LIST: -0.1 (-0.6 to 0.4) |  |  |
| LSR: -0.4 (-0.8 to 0.0) | -0.3 (-1.0 to 0.3) | -0.4 (-1.2 to 0.4) |
| Sleepiness |  |  |
| MBSR: -0.7 (-1.3 to -0.0) | -0.3 (-1.1 to 0.5) | -0.3 (-1.1 to 0.5) |
| WAITING LIST: -0.4 (-0.9 to 0.1) |  |  |
| LSR: -0.1 (-0.6 to 0.4) | 0.3 (-0.4 to 1.0) | 0.3 (-0.5 to 1.1) |
| Comfort |  |  |
| MBSR: 0.8 (0.3 to 1.3) | 0.5 (-0.2 to 1.2) | 0.5 (-0.2 to 1.3) |
| WAITING LIST: 0.3 (-0.3 to 0.8) |  |  |
| LSR: 0.5 (0.1 to 0.9) | 0.2 (-0.4 to 0.9) | -0.1 (-0.9 to 0.7) |
| Somatic Awareness |  |  |
| MBSR: 0.5 (0.0 to 0.9) | 0.5 (-0.1 to 1.1) | 0.6 (0.02 to 1.2) |
| WAITING LIST: -0.1 (-0.5 to 0.4) |  |  |
| LSR: 0.6 (0.1 to 1.0) | 0.6 (-0.0 to 1.2) | 0.4 (-0.3 to 1.0) |
|  |  |  |

RCT: Randomised controlled trial; MBSR: Mindfulness Based Stress Reduction; LSR: Locally developed stress reduction intervention; CI: confidence interval; PSS: Perceived Stress Scale; SCL-5: Hopkins Symptom Check List-5; WHO-5: WHO-5-wellbeing scale; BRS: Brief Resilience Scale; ARSQ: The Amsterdam Resting State Questionnaire; SCS: Self-Compassion Scale; FFMQ: The Five Facet Mindfulness Questionnaire; EQ: Experiences Questionnaire - Decentering sub scale ^a^Adjusted for age, sex, educational level, history of mental disorder, baseline PSS, SCL-5, WHO-5, BRS
