# Supplementary material for: A pilot randomised trial comparing a mindfulness-based stress reduction course, a locally-developed stress reduction intervention and a waiting list control group in a real-life municipal health care setting
Source: BMC Public Health. 2020 Mar 30;20:409. doi: 10.1186/s12889-020-08470-6 (PMC7106861; doi:10.1186/s12889-020-08470-6)
Supplement: Supplementary file 2 — Additional file 2: Table 2a. (Proposed mediators) Indications of effectiveness of MBSR compared with LSR 12 weeks from baseline (regression analysis). A three-armed pilot RCT among individuals seeking help due to stress in a Danish Municipal Health Care Center (19 + 18 in the MBSR and LSR group, respectively), 2018. [file 12889_2020_8470_MOESM2_ESM.docx]

**Table 2a (Proposed mediators)** Indications of effectiveness of MBSR compared with LSR 12 weeks from baseline (regression analysis). A three-armed pilot RCT among individuals seeking help due to stress in a Danish Municipal Health Care Center (19+18 in the MBSR and LSR group, respectively), 2018.

| Change 12 weeks from  baseline,  mean (95%CI) | Difference, mean (95%CI) | Adjusted^a^ difference, mean (95%CI) |
| --- | --- | --- |
| *Self-reported outcomes (score-points)* |  |  |
| Mindfulness (FFMQ_15) |  |  |
| MBSR: 6.1 (1.5 to 10.7) | 0.3 (-5.7 to 6.4) | -0.4 (-7.3 to 6.6) |
| LSR: 5.8 (1.8 to 9.8) |  |  |
| Self-Compassion (SCS) |  |  |
| MBSR: 7.1 (2.7 to 11.4) | 0.3 (-5.4 to 6.0) | 1.1 (-5.8 to 8.1) |
| LSR: 6.7 (2.8 to 10.7) |  |  |
| Decentering (EQ) |  |  |
| MBSR: 6.9 (2.4 to 11.5) | 3.5 (-2.5 to 9.4) | 6.6 (-0.5 to 13.7) |
| LSR: 3.5 (-0.3 to 7.4) |  |  |
| *Amsterdam Resting-State Questionnaire* |  |  |
| Discontinuity of mind |  |  |
| MBSR: -1.2 (-1.7 to -0.7) | -0.4 (-1.1 to 0.2) | -0.6 (-1.3 to 0.1) |
| LSR: -0.8 (-1.2 to -0.3) |  |  |
| Theory of mind |  |  |
| MBSR: -0.1 (-0.5 to 0.3  LSR: -0.2 (-0.7 to 0.3) | 0.1 (-0.5 to 0.7) | -0.1 (-0.6 to 0.7) |
| Self |  |  |
| MBSR: -0.3 (-0.8 to 0.2)  LSR: -0.2 (-0.6 to 0.3) | -0.1 (-0.8 to 0.6) | -0.3 (-1.0 to 0.4) |
| Planning |  |  |
| MBSR: -1.0 (-1.5 to -0.5) | -0.6 (-1.2 to 0.0) | -0.8 (-1.5 to -0.1) |
| LSR: -0.4 (-0.8 to 0.0) |  |  |
| Sleepiness |  |  |
| MBSR: -0.7 (-1.3 to -0.0) | -0.6 (-1.4 to 0.2) | -1.0 (-1.7 to -0.2) |
| LSR: -0.1 (-0.6 to 0.4) |  |  |
| Comfort |  |  |
| MBSR: 0.8 (0.3 to 1.3) | 0.3 (-0.3 to 0.9) | 0.2 (-0.4 to 0.9) |
| LSR: 0.5 (0.1 to 0.9) |  |  |
| Somatic Awareness |  |  |
| MBSR: 0.5 (0.0 to 0.9) | -0.1 (-0.7 to 0.5) | -0.04 (-0.7 to 0.7) |
| LSR: 0.6 (0.1 to 1.0) |  |  |

RCT: Randomised controlled trial; MBSR: Mindfulness Based Stress Reduction; LSR: Locally developed stress reduction intervention; CI: confidence interval; PSS: Perceived Stress Scale; SCL-5: Hopkins Symptom Check List-5; WHO-5: WHO-5-wellbeing scale; BRS: Brief Resilience Scale; ARSQ: The Amsterdam Resting State Questionnaire; SCS: Self-Compassion Scale; FFMQ: The Five Facet Mindfulness Questionnaire; EQ: Experiences Questionnaire - Decentering sub scale ^a^Adjusted for age, sex, educational level, history of mental disorder, baseline PSS, SCL-5, WHO-5, BRS
